# Chinese Physicians’ Preference for Prescribing Brand-Name vs. Generic: A Discrete Choice Experiment

**DOI:** 10.34172/ijhpm.8392

**Published:** 2024-12-29

**Authors:** Ruilin Wang, Zhiyuan Wang, Xiaoyu Li, Lin Bai, Pingan Fan, Huangqianyu Li, Yuanyuan Tang, Xin Li, Yangmu Huang, Xiaoyan Nie, Luwen Shi, Jing Chen

**Affiliations:** ^1^Department of Pharmacy Administration and Clinical Pharmacy, School of Pharmaceutical Sciences, Peking University, Beijing, China.; ^2^International Research Center for Medicinal Administration, Peking University, Beijing, China.; ^3^Bidding Management Office, Suqian First Hospital, Suqian, China.; ^4^Department of Clinical Pharmacy, School of Pharmacy, Nanjing Medical University, Nanjing, China.; ^5^School of Public Health, Peking University, Beijing, China.

**Keywords:** Prescribing Behavior, Generic Drugs, The Consistency Evaluation Policy, Discrete Choice Model, Influencing Factors

## Abstract

**Background:**

Promoting the use of generic drugs is a viable strategy to control drug costs. As physicians have a critical role in deciding on what drugs to prescribe and thus whether certified generic drugs were actually used, this study aimed to analyze factors influencing whether physicians were willing to prescribe generic drugs versus brand-name drugs after the implementation of Consistency Evaluation Policy (CEP).

**Methods:**

A discrete choice experiment (DCE) was developed to explore factors influencing physicians’ preferences toward prescribing brand-name drugs versus its certified generic. There were four attributes in the model, namely prices, hospital-level cost control measures, information about clinical safety and efficacy of generic drugs, and reimbursement rate. In total, 1297 physicians from 101 hospitals participated in the study and 1047 questionnaires were retained.

**Results:**

We found that substantial disclosed information about the generic’s clinical safety and efficacy (Sufficient information, odds ratio [OR]=3.251, 95% CI=3.098-3.412), lower price of the certified generic (price ratio of generic drugs versus brand-name drugs=1: 10, OR=1.130, 95% CI=1.078-1.185), stringent hospital cost control measures (the brand-name drugs were affected by the national centralized drug procurement (NCDP) policy or a tight cost-control measure, OR=1.247, 95% CI=1.190-1.307), and lower reimbursement rates for brand-name drugs (reimbursement rate=20%, OR=1.283, 95% CI=1.224-1.346) all increased physicians’ propensity to prescribe certified generic drugs.

**Conclusion:**

When certified generic drugs were lower priced or disclosed more information about their clinical safety and efficacy or when brand-name drugs were subject to tighter hospital cost control measures, physicians were more inclined to prescribe certified generic drugs. The findings suggest that CEP, together with the NCDP to promote market competition and hospital cost control measures targeting brand-name drugs, promoted the use of generic drugs through influencing physician prescribing behavior. The widely use of generic drugs may further benefit from increased disclosure of the clinical safety and efficacy of generic drugs by their manufacturers.

## Introduction

**Key Messages**

**Implications for policy makers**

- Our results illustrated that price, disclosed information about the generic drugs’ clinical safety and efficacy, hospital-level cost control measured targeting brand-name drugs, and differential reimbursement rates influenced whether physicians were willing to prescribe generic drugs.
- The findings suggest that Consistency Evaluation Policy (CEP), together with the national centralized drug procurement (NCDP) to promote market competition and hospital cost control measures targeting brand-name drugs, promoted the use of generic drugs through influencing physician prescribing behavior. The widely use of generic drugs may further benefit from increased disclosure of the clinical safety and efficacy of generic drugs by their manufacturers.

**Implications for the public**

For most developing countries, promoting the use of generic drugs is a viable strategy to satisfy basic medical needs while containing expenditures on prescription drugs. The public and healthcare workers may be skeptical about the efficacy of generic drugs, partly due to limited knowledge about generic drugs and their regulation. Physicians hold a critical role in deciding on what drugs to prescribe, factors influencing their prescribing preferences between brand-name drugs versus their generic drugs need to be explored to fully promote the use of generic drugs, alongside efforts to strengthen the quality of generic drugs on the market.

Rising healthcare expenditure is a common public health challenge globally. Promoting the use of generic drugs can help save on drug expenditures and is an effective strategy to control medical costs.^1-6^ Due to factors such as history, concept and technology, China did not implement strict quality standards for generic drugs in the early days, resulting in the lack of competitiveness of generic drugs, low recognition of generic drugs by patients, and the brand-name drugs can maintain a high price even if the patent expires.^7^ Hence, improving the public’s knowledge of and confidence in generic drugs is critical to promoting the use of generic drugs.^8^ Previous studies have found that insufficient disclosure of clinical safety and efficacy of the generic drugs and concerns about the difference in incidences of adverse events attributable to generic versus brand-name drugs may still hold physicians back from prescribing generic drugs.^9-14^ Understanding factors influencing physicians’ prescribing behaviors is pivotal to promoting the use of generic drugs.^15^

 China has faced challenges arisen from satisfying basic medical needs of a vast population with a limited budget and has taken multiple actions to promote the use of generic drugs by improving their quality and lowering their price. In the past, the absence of requirements to assure comparable efficacy and safety profile of generic drugs with their brand-name drugs in China had led to the launch of low-quality generic drugs and a general mistrust of physicians and patients about the quality of generic drugs.^16,17^ To address this problem, China introduced the Consistency Evaluation Policy (CEP) in 2012 that certifies high-quality generic drugs (ie, generic drugs that have comparable efficacy and safety profiles to their brand-name counterparts) to improve the overall quality of generic drugs, rebuild the public’s confidence, and promote the use of generic drugs.^18-22^

 However, the views of physicians about prescribing generic drugs remain unclear after the implementation of CEP.^8,23-26^ This study aimed to analyze the factors influencing physicians’ preferences of prescribing generic versus brand-name drugs and explore the influence of other policy initiatives on the use of generic drugs. This study could suggest possible policy initiatives to develop to further promote the use of generic drugs for other developing countries.

## Methods

###  Attribute and Level Identification

 Some factors would have a significant influence on physicians’ prescription behavior and have been illustrated in previous studies. Many outer settings would contribute to the difference of prescription behavior, including differing reimbursement regimens for different drugs and influence from pharmaceutical industry.^27^ All these outer settings would affect the prescription behavior based on effectiveness, safety and cost-effectiveness of drugs, the most important factor that would influence the prescription behavior in China is the effectiveness of drugs, because physicians would always choose some drugs that often used in clinic, which could help them ensure that these drugs are stable and effective, therefore, previous medication status and the safety and efficacy information disclosed by pharmaceutical industry would be an important factor in prescription behavior.^28,29^ Also, when physicians prescribing drugs, they have to consider the financial status of patients, especially for those patients with low income and reimbursement ratios.^28,30-32^ Therefore, physicians prescription behavior would influenced by the price and reimbursement rates of brand-name drug and its generic drugs, Chinese government has launched the national centralized drug procurement (NCDP) to promote the use and control the costs of generic drugs, the process of centralized drug procurement in China included bidding, purchasing and use, and only those drug manufacturers passing the generic drugs consistency evaluation are qualified to the bidding, which would motivate more generic drug manufacturers to participate in consistency evaluation, and the quality and efficacy of generic drug in China would be improved gradually.^33,34^

 Physicians’ knowledge and beliefs would have an influence on prescription behavior, especially for those physicians with many years clinical practice experience and education that would help them form their own preference of prescription based on their past prescription behavior and experience.^27,35,36^ Based on theory of planned behavior, physicians’ prescription behavior would also influence by physicians’ attitude, subjective norms, perceived behavioral control, behavioral intention and actual behavior,^37,38^ and these inner factors of physicians’ preference towards generic prescribing behavior have been discussed in our previous study.^39^

 To explore physicians’ preferences of prescribing brand-name drugs versus generic drugs, a series of scenarios were developed using pairs of specific brand-name drugs (Drug A) and generic drugs certified by the CEP (Drug B) as alternatives in the discrete choice experiment (DCE). The main outcome was the physician’s choices in different scenarios, if generic drugs were selected, y = 1, while if brand-name drugs were selected, y = 0. First, we assigned Drug B a price lower than Drug A. The price of Drug B was set based on the procurement price of drugs that passed the NCDP program. Based on expert interviews and the healthcare landscape in China, we selected three representative levels—low, medium, and high—for both price ratios and reimbursement ratios to investigate how different settings influence physicians’ prescribing preferences.^40-43^ The price ratio of certified generic versus brand-name drug was set at the followings: (1) 1:10, (2) 3:10, and (3) 1:2.^44^ Second, to explore the impact of reimbursement rate on physicians’ prescribing behavior, the reimbursement rate of Drug B was set at 90%, while the reimbursement rate for Drug A was assigned one of the following scenarios: (1) 20%, (2) 50%, or (3) 80%. Third, as difference in the levels of disclosure of clinical safety and efficacy between Drug A and Drug B also affected physicians’ prescribing behavior, Drug A was set as having disclosed substantial information about clinical safety and efficacy (as expected for a pioneer drug) and information for Drug B was assigned one of the following scenarios: (1) substantial information on clinical safety and efficacy; (2) insufficient information on clinical safety and efficacy; or (3) no information available. Fourth, considering that some hospitals take cost control measures targeting the use of brand-name drugs to control drug costs and improve overall efficiency, the following scenarios of cost control measures were applied to Drug A sequentially: (1) not restricted by any hospital-level cost control measures; (2) affected by hospital-level cost control measures; and (3) affected by NCDP and thus patients need to acquire Drug A out-of-hospital.

 As the selection of drugs to prescribe would be affected by the patient’s socioeconomic status and medical condition,^45^ we conducted three subgroup analyses to explore the influence of patients’ health insurance status, previous medication status, and severity of illness on physicians’ prescribing preferences.^46-48^ Factors such as education level, clinical practice experience, and gender of physicians may also have an impact on prescribing behavior. Therefore, these factors were included in the model as covariates.^49-53^ The rationale for selecting variables is was shown in Table 1.

**Table 1:** Rationale of Variables in the Discrete Choice Experiment of Physicians’ Preferences of Prescribing Brand-Name Drugs Versus Generic Drugs

**Variable**	**Rationale**
Price of certified generic drug prices vs. its brand-name drugs	As launching a brand-name drug entails extensive time and financial investments, a brand-name drug is almost always highly priced. Launching generic drugs requires less investments, which enable them to be priced much lower than the brand-name drug. Numerous studies have demonstrated that difference in the price of brand-name drugs versus generic drugs heavily influences physicians’ prescribing behavior.^15,21,54-56^
Hospital-level cost control measures targeting brand-name drugs	In China, hospitals set a department to manage issues concerned with healthcare insurance and reimbursement. The department could request healthcare providers to adjust their prescribing (eg, prioritize prescribing certain generic drugs) according to the cost control measures it developed to control expenditures on brand-name drugs.^57,58^
Information about generic drugs’ clinical safety and efficacy	Physicians and patients could determine whether generic drugs are used in clinical practice. It has been found that information about drugs’ clinical safety and efficacy could impact the physician’s prescribing behavior.^59-62^
Reimbursement rates of brand-name drugs	Physicians’ prescribing behavior are affected by the patient's socioeconomic status and medical condition. The difference in medical insurance reimbursement rate of different drugs also affects physicians' prescribing behavior.^46-48^

###  Design of Discrete Choice Experiment

 Each of the four attributes (price, hospital-level cost control measure, disclosed information about clinical safety and efficacy, and reimbursement rate) had three thresholds and therefore together their combinations generated 81 scenarios. Based on the orthogonal main effect design, nine scenarios were incorporated into this questionnaire (Table S1, Supplementary file 1). Physicians who participated in the questionnaire were required to choose between Drug A and Drug B in each scenario, depending on the patient’s health insurance status, previous medication status, and severity of illness^63^ (Table 2). All participants provided informed consent.

**Table 2:** Description of Attributes and Levels in the Discrete Choice Experiment of Physicians’ Preferences of Prescribing Brand-Name Drugs Versus Generic Drugs

**Attributes**	**Attributes Level**	
Drug	Brand-name drug (Drug A)	High-quality generic drug (Drug B)
Drug prices	A_1_	B_1_ B_2_ B_3_
Reimbursement rates	20% 50% 80%	90%
Hospital cost control measures	Not restricted by hospital cost control measures Affected by hospital cost control measures Affected by the NCDP policy	Not restricted by hospital cost-controls
Information about clinical safety and efficacy	Sufficient information	Sufficient information Insufficient information No information available
Health insurance status	With health insurance Without health insurance	With health insurance Without health insurance
Previous medication status	First-visit patients Patients who previously used brand-name drugs Patients who previously used generic drugs	First-visit patients Patients who previously used brand-name drugs Patients who previously used generic drugs
Severity of illness	Mild and moderate disease Severe disease	Mild and moderate disease Severe disease

Abbreviation: NCDP, national centralized drug procurement.

###  Target Population and Sample Size

 Participants were enrolled if they (1) were a licensed physician; (2) had been prescribing for more than a year; (3) practiced at sample healthcare institutions; and (4) were a native Chinese speaker. No incentives were offered to the enrolled participants. A convenience sampling method was used to distribute the questionnaire at the 101 sample hospitals. We have calculated the minimum sample size for our DCE study according to the guideline,^64^ and the R-code have been added in Supplementary file 2.

###  Data Collection

 The questionnaire was first piloted in 2019 and the formal questionnaire was conducted in 2020. We designed the questionnaire-based literature and stakeholder interviews. The initial questionnaire was piloted among a sample of 116 physicians to ensure feasibility and optimal ordering of questions. Based on the results of the 116 initial questionnaires, we found that physicians who carefully fill out the questionnaire will take no less than 5 minutes to complete, therefore, we set the minimum response time as 5 minutes to avoid low-quality responses. Results generated from the pilot questionnaire were not included in the analysis. After minor modifications, the final version of the questionnaire, provided in the Supplement, was composed of two sections: (1) participant demographics and (2) the DCE involving the nine selected scenarios. Questionnaire that was returned in printed version were entered into a Microsoft Excel spreadsheet while the electronic questionnaire was powered by https://www.wjx.cn. If the surveyed physician chose Drug A or Drug B in all scenarios or that he/she failed to answer all questions, the questionnaire was excluded from the statistical analysis to produce meaningful results in the logistic regression. The data were compiled in Microsoft Excel and validated for errors.

 In total, there were 1297 physicians participated in the study. There were 35 questionnaires incomplete and thus excluded from the final analysis and another 215 questionnaires were excluded as the response time was lower than the minimum required (which might imply that these were low-quality responses). Thus, data analyses included 1047 (80.7%) participants from 101 hospitals, which exceeded the minimum sample size required for our DCE design. Participants’ gender was approximately even (50.2% were male and 49.8% were female); 22.9% of participants attained a PhD degree, 43.2% a master’s degree, and 33.9% a bachelor’s degree or less. Most surveyed physicians worked at tertiary hospitals (75.8%). Two-thirds of surveyed physicians (66.1%) had been in clinical practice for 1-10 years, 22.5% for 11-20 years, and 11.4% for more than 20 years (Table 3).

**Table 3:** Sample Characteristics of Surveyed Physicians

**Characteristics**	**Frequency**	**Percent**
Gender		
Female	521	49.80
Male	526	50.20
Years of clinical practice of physicians		
≤10	692	66.10
(10, 20]	236	22.50
＞20	119	11.40
Highest educational attainment of physicians		
Undergraduate and below*	355	33.90
Master	452	43.20
PhD	240	22.90
Hospital level		
Tertiary	794	75.80
Secondary	233	22.30
Primary	20	1.90
Hospital location		
Jiangsu	565	54.00
Beijing	294	28.10
Guangdong	76	7.30
Xinjiang	73	7.00
Fujian	39	3.70
Specialty		
Cardiology	92	8.79
Infectious diseases	20	1.91
Endocrinology	41	3.92
Respiration	53	5.06
Gastroenterology	37	3.53
Nephropathy	22	2.10
Oncology	45	4.30
Hematology	31	2.96
Neurology	59	5.64
Internal medicine	24	2.29
Thoracic surgery	23	2.20
Urology	27	2.58
Neurosurgery	29	2.77
Orthopedics	66	6.30
General surgery	53	5.06
Gynecology	43	4.11
Obstetrics	27	2.58
Pediatrics	58	5.54
Dermatology	23	2.20
Emergency	32	3.06
Psychiatry	13	1.24
Others	229	21.87

* In China, according to Laws, after accumulating enough clinic practice experience, and passed the Examination for the Qualifications of Licensed Doctor, would be qualified as a physician or a prescriber.

###  Statistical Analysis

 As DCE measures choices made by the same physician in different scenarios and data from the same physician might be inter-correlated, we conducted a mixed-effects logistic regression analysis to explore the impact of influencing factors on physician prescribing behaviors.^65,66^ The mixed-effects logistic regression approach does not require functional transformation of the raw frequency counts that may or may not succeed in rendering a normal distribution.^65^ We conducted descriptive analyses of the physicians’ characteristics. The primary analysis consisted of the variables involved in the DCE design and the subgroup analysis involved patients’ health insurance status, prior medication status, and disease severity. All attributes were included as categorical variables. The Akaike information criterion and Bayesian information criterion were used in model selection.^67^ All statistical tests performed were 2-sided and a *P* < .05 was considered statistically significant. All analyses were conducted by two investigators with Stata (version 15; Stata Corp LLC).

## Results

###  Primary Analysis

 Lower price or substantial disclosed information about clinical safety and efficacy of Drug B were associated with more physician willingness to prescribe it (price ratio of Drug B: Drug A = 1:10, odds ratio [OR] = 1.130, 95% CI = 1.078-1.185; sufficient information of Drug B, OR = 3.251, 95% CI = 3.098-3.412). Meanwhile, lower reimbursement rates of or more stringent cost control measures targeting Drug A was associated with higher likelihood of physicians prescribing Drug B (reimbursement rate of Drug A = 20%, OR = 1.283, 95% CI = 1.224-1.346; Drug A was affected by hospital-level cost control measure, OR = 1.156, 95% CI = 1.103-1.212; Drug A was affected by the NCDP policy, OR = 1.247, 95% CI = 1.190-1.307). Besides, physicians with more years of clinical practice were significantly more likely to prescribe Drug B (more than 20 years of clinical practice, OR = 2.173, 95% CI = 1.410-3.350) while physicians with higher educational attainment were significantly less likely to prescribe Drug B (attained a PhD degree, OR = 0.686, 95% CI = 0.480-0.982) (Table 4, Figure S1).

**Table 4:** Primary Analysis of Physicians’ Preferences for Prescribing Brand-Name Drugs and Generic Drugs

**Primary Analysis**	**OR**	***P***	**Standard Error**	**95% CI**	
Price ratio of generic drugs vs. brand-name drugs					
1:2	Reference				
3:10	1.155	***	0.028	1.102	1.210
1:10	1.130	***	0.027	1.078	1.185
Hospital cost control measures targeting brand-name drugs					
Not restricted by hospital cost controls	Reference				
Affected by hospital cost control measures	1.156	***	0.028	1.103	1.212
Affected by the NCDP policy	1.247	***	0.030	1.190	1.307
Information about clinical safety and efficacy of generic drugs					
No information available	Reference				
Insufficient information	1.121	***	0.027	1.070	1.175
Sufficient information	3.251	***	0.080	3.098	3.412
Reimbursement rates of brand-name drugs					
80%	Reference				
50%	1.039	-	0.025	0.991	1.089
20%	1.283	***	0.031	1.224	1.346
Years of clinical practice					
≤10	Reference				
(10, 20]	1.367	-	0.224	0.990	1.884
>20	2.173	***	0.480	1.410	3.350
Highest educational attainment of physicians					
Undergraduate and below	Reference				
Master	0.638	**	0.099	0.471	0.864
PhD	0.686	*	0.126	0.480	0.982
Gender					
Female	Reference				
Male	1.220	-	0.166	0.935	1.593

Abbreviations: NCDP, national centralized drug procurement; OR, odds ratio; CI, confidence interval. * *P* ≤ .05, ** *P* ≤ .01, *** *P* ≤ .001, Brand-name drugs choice = 0, High-quality generic drugs choice = 1.

###  Subgroup Analyses

 Physicians were less likely to prescribe Drug B for patients with a health insurance than without one (Drug A’s reimbursement rate was at 20%, 50%, and 80%, OR = 0.850, 0.520, and 0.501, respectively) (Table S2, Figure S3). Physicians were more likely to prescribe Drug A for patients who previously used brand-name drugs (OR = 0.279, 95% CI = 0.256-0.303) while they were also more likely to prescribe Drug B for patients who previously used generic drugs (OR = 4.836, 95% CI = 4.444-5.264) (Figure S2, Table S3, and Figure S4). For patients with a severe disease, physicians were significantly less likely to prescribe Drug B (OR = 0.070, 95% CI = 0.063-0.078) (Table S4, Figure S5, Table 5).

**Table 5:** Subgroup Analysis of Physicians’ Preferences for Prescribing Brand-Name Drugs and Generic Drugs

**Subgroup Analysis**	**OR**	***P***	**Standard Error**	**95% CI**	
Group A: Reimbursement rates of brand-name drugs* Health insurance status					
Without health insurance	Reference				
80%	0.501	**	0.029	0.447	0.562
50%	0.520	***	0.030	0.465	0.581
20%	0.850	***	0.049	0.760	0.951
Group B: Previous medication status					
First-visit patients	Reference				
Patients who previously used brand-name drugs	0.279	***	0.012	0.256	0.303
Patients who previously used generic drugs	4.836	***	0.209	4.444	5.264
Group C: Severity of illness					
Mild and moderate disease	Reference				
Severe disease	0.070	***	0.004	0.063	0.078

Abbreviations: NCDP, national centralized drug procurement; OR, odds ratio; CI, confidence interval. * *P* ≤ .05, ** *P* ≤ .01, *** *P* ≤ .001, Brand-name drugs choice = 0, High-quality generic drugs choice = 1. Complete subgroup analysis results and questionnaire design of our study can be found in Supplementary files 1 and 3.

## Discussion

 We found that substantial disclosed information about the certified generic’s safety and efficacy information, lower price of the certified generic in relative to its brand-name drug, tight hospital cost control measure targeting brand-name drugs, and lower reimbursement rates for brand-name drugs all increased physicians’ propensity to prescribe certified generic drugs. Patients’ health insurance status, previous medications used, and severity of illness also had significant effects on physicians’ prescribing preferences. Physicians were more likely to prescribe the brand-name drugs for patients who previously used them than those who had not used. Their prescribing preference was also influenced by the patient’s previous medication status and might reflect the influence of factors such as medication inertia and patient willingness.^68,69^

 The key to promoting the use of generic drugs is to convince the physicians that generic drugs are therapeutically equivalent to brand-name drugs. A previous study found that generic switching was associated with poorer clinical outcomes and more adverse events.^69^ For drugs in specific therapeutic areas such as endocrinology and epilepsy, differences in guideline recommendations for switching between generic and brand-name drugs may also influence patients’ medication use.^70,71^ Hence, the absence of information about the generic drugs’ clinical safety and efficacy, particularly for drugs to treat serious conditions, may give rise to concerns and refrain physicians from prescribing generic drugs.^9,72-75^ Although the consistency evaluation of generic drugs is ongoing, due to physicians’ insufficient understanding of CEP and their subjective clinical experience, the concept of “poor efficacy” of generic drugs cannot be completely reversed in the short term, and physicians generally lack confidence in generic drugs.^76^ A survey of physicians and pharmacists shows that most professionals have a positive attitude toward generic drugs substitution, but about one-third of respondents remain neutral about the clinical equivalence of generic drugs with brand-name drugs and generic drug substitution when it comes to safety and efficacy.^77^ In addition, patients’ knowledge, attitudes, and trust in generic drugs can influence physicians’ prescribing behavior.^78^ Therefore, enhancing research on the clinical efficacy of generic drugs, disclosing the generic drugs’ clinical safety and efficacy and increasing physician and patient understanding of generic drugs and CEP can help promote the use of generic drugs. Prescribing behavior is also affected by the cost of the drugs. As brand-name drugs are typically more expensive, promoting the use of generic drugs may reduce healthcare expenditures.^79^ Previous studies also found that increased competition amongst different generic products in the same class tended to further decrease drug prices.^80^ In addition, creating groups of drug procurement across administrative areas could increase their bargain power to further lower drug prices, which is a strategy implemented in a few countries.^81^ The NCDP requires that physicians give priority to generic drugs procured through the program when prescribing, which has promoted the use of generic drugs to reduce expenditures. Similarly, strict hospital-level cost control measure targeting brand-name drugs have been used to promote the prescribing of the selected generic drugs,^82,83^ all of these initiatives can affect the likelihood of physicians prescribing certified generic drugs.^17,44,84^

 Besides, payments may influence physicians’ clinical decision-making and drug prescribing behavior, adjusting reimbursement rates may incentivize physicians to prescribe certain drugs, which is an important leverage point to limit the over-prescribing of higher-priced brand-name drugs.^79,85^ The performance appraisal system and the diagnosis-related group-based case-mix payment system was implemented in China to control the rising medical costs and improve efficiency of the payment system.^86,87^ Therefore, the public hospital limited the use of brand-name drugs through hospital cost-control constraints to promote the use of certified generic drugs and improve overall system efficiency. In this study, we found that when hospitals had cost control measures targeting brand-name drugs (ie, physician need to limit the prescribing of those drugs), the probability that physicians prescribe certified generic drugs would increase significantly, which is similar to the effect of some generic substitution measures in the United States.^1,88^ Drug budgets could be set at the physician-level to gradually adjust physicians’ prescribing preferences and thus behaviors.

 Consistent with an existing study,^8^ we found that young, highly-educated physicians had a lower propensity to prescribe generic drugs. A previous study suggested that physicians’ attitude toward and knowledge of the use of generic drugs had a positive influence on generic prescription behavior.^89^ Studies also have shown that education intervention can be a critical tactic to increase physicians knowledge of and confidence in prescribing generic drugs.^90^ Developing advocacy programs about the use of generic drugs for young, highly-educated physicians may help to adjust their prescribing behaviors.^91^

 We designed a DCE to explore the influence of factors associated with brand-name drugs versus their certified generic, patients, and policy on physicians’ prescribing behaviors. Our findings illustrate Chinese physicians’ preference for prescribing behavior and analyze influencing factors, for many developing countries, the findings of our study would help to develop relative drug policy (information disclosure, price and reimbursement) to further improve the use of generic drugs and reduce healthcare expenditures. However, there are still some limitations of this study when interpreting our results. First, we did not analyze the actual prescribing behaviors of physicians and there might be discrepancies between physicians’ propensities and their actual behaviors. Further validation of the study results is needed by examining prescriptions they actually prescribe. Besides, thresholds set for the four attributes involved in the experiment were defined based on the differences between certified generic drugs and brand-name drugs, which may differ from the real-world situation. However, these deviations are moderate as we strived to follow the actual scenario and standardized questionnaire design.

## Conclusion

 Our findings provide important insights into factors influencing physicians’ prescribing preferences between brand-name drug versus its certified generic after the implementation of CEP. Price, information about the generic drugs’ clinical safety and efficacy, hospital cost control measures, and medical insurance reimbursement rates are all important factors influencing physicians’ prescribing preferences. Results suggest that setting hospital cost control measures targeting brand-name drugs and requiring generic manufacturers proactively to disclose information about the clinical safety and efficacy of generic drugs may help to promote the use of generic drugs. This will need cross-sectional collaboration of concerned national agencies, medical institutions, and manufacturers.

## Acknowledgments

 We would like to express our gratitude to all physicians who responded to our questionnaire. We also feel grateful for Dr. Frances J. Richmond and Dr. C. Benson Kuo from University of Southern California for help with editing.

## Ethical issues

 The study protocol was approved by the Peking University Medical Ethics Committee (IRB00001052–19026). All participants provided written informed consent.

## Conflicts of interest

 Authors declare that they have no conflicts of interest.

## Data availability statement

 The datasets generated for this study are available on request to the corresponding author.

## Supplementary files

Supplementary file 1. contains Tables S1-S5 and Figures S1-S5.

Supplementary file 2. Sample Size DCE.R.

Supplementary file 3. The Questionnaire of Our Study.
