# Supplementary material for: Chinese Physicians’ Preference for Prescribing Brand-Name vs. Generic: A Discrete Choice Experiment
Source: Int J Health Policy Manag. 2024 Dec 29;13:8392. doi: 10.34172/ijhpm.8392 (PMC11806230; doi:10.34172/ijhpm.8392)

Article title: Chinese Physicians' Preference for Prescribing Brand-Name vs. Generic: A Discrete Choice Experiment

Journal name: International Journal of Health Policy and Management (IJHPM)

Authors' information: Ruilin Wang[1]¶, Zhiyuan Wang[1]¶, Xiaoyu Li[1], Lin Bai[1], Pingan Fan[1], Huangqianyu Li[2], Yuanyuan Tang[3], Xin Li[4], Yangmu Huang[5], Xiaoyan Nie[1,2], Luwen Shi[1,2], Jing Chen[1,2]*

[1]Department of Pharmacy Administration and Clinical Pharmacy, School of Pharmaceutical Sciences, Peking University, Beijing, China.

[2]International Research Center for Medicinal Administration, Peking University, Beijing, China.

[3]Bidding Management Office, Suqian First Hospital, Suqian, China.

[4]Department of Clinical Pharmacy, School of Pharmacy, Nanjing Medical University, Nanjing, China.

[5]School of Public Health, Peking University, Beijing, China.

***Correspondence to:** Jing Chen; Email: jingchen@bjmu.edu.cn

¶ Both authors contributed equally to this paper.

Citation: Wang R, Wang Z, Li X, et al. Chinese physicians' preference for prescribing brand-name vs. generic: a discrete choice experiment. Int J Health Policy Manag. 2024;13:8392. doi:10.34172/ijhpm.8392

Supplementary file 1

Table S1. Selected 9 scenarios based on orthogonal main effect design

Variable	High-quality generic drug prices vs. Originator drug prices	Hospital cost control constraints of originator drugs	Information on clinical safety and efficacy of generic drugs	Reimbursement rates of originator drugs
Scenario 1	1:2	Not restricted by hospital cost controls	Sufficient information	50%
Scenario 2	3:10	Affected by hospital cost control	No information available	50%
Scenario 3	1:10	Affected by hospital cost control	Sufficient information	20%
Scenario 4	3:10	Affected by the NCDP policy	Sufficient information	80%
Scenario 5	3:10	Not restricted by hospital cost controls	Insufficient information	20%
Scenario 6	1:2	Affected by hospital cost control	Insufficient information	80%
Scenario 7	1:2	Affected by the NCDP policy	No information available	20%
Scenario 8	1:10	Not restricted by hospital cost controls	No information available	80%
Scenario 9	1:10	Affected by the NCDP policy	Insufficient information	50%

S2. Subgroup analysis of health insurance status of physicians' preferences for prescribing originator and its generic drugs

Subgroup analysis A	Odds Ratio	P	Std. Err.	95% Conf.Interval	
Reimbursement rates of originator drugs*Health insurance status					
Without health insurance	Reference				
80%	0.501	**	0.029	0.447	0.562
50%	0.520	***	0.030	0.465	0.581
20%	0.850	***	0.049	0.760	0.951
Price ratio of generics vs. originator drugs					
1:2	Reference				
3:10	1.180	***	0.058	1.071	1.299
1:10	1.138	**	0.056	1.033	1.254
Hospital cost control constraints targeting originator drugs					
Not restricted by hospital cost controls	Reference				
Affected by hospital cost control measures	1.205	***	0.060	1.093	1.328
Affected by the NCDP policy	1.252	***	0.062	1.136	1.379
Information about clinical safety and efficacy of generic drugs					
No information available	Reference				
Insufficient information	1.182	***	0.057	1.074	1.300
Sufficient information	6.019	***	0.318	5.427	6.675
Years of clinical practice					
≤10	Reference				
(10, 20]	1.629	*	0.321	1.107	2.398
>20	2.646	***	0.703	1.572	4.445
Highest education attainment of physicians					
Undergraduate and below	Reference				
Master	0.672	*	0.125	0.466	0.969
PhD	0.663		0.146	0.431	1.022
Gender					
Female	Reference				
Male	1.406	*	0.230	1.021	1.937

(Note)*P ≤ 0.05, **P ≤ 0.01, ***P ≤ 0.001, Originator choice = 0, High-quality generic choice =1

S3. Subgroup analysis of previous medication status of physicians' preferences for prescribing originator and its generic drugs

Subgroup analysis B	Odds Ratio	P	Std. Err.	95% Conf.Interval	
Previous medication Status					
First-visit patients	Reference				
Patients who previously used originator drugs	0.279	***	0.012	0.256	0.303
Patients who previously used generic drugs	4.836	***	0.209	4.444	5.264
Price ratio of generics vs. originator drugs					
1:2	Reference				
3:10	1.238	***	0.052	1.141	1.343
1:10	1.215	***	0.051	1.119	1.319
Hospital cost control constraints targeting originator drugs					
Not restricted by hospital cost-controls	Reference				
Affected by hospital cost-control measures	1.260	***	0.053	1.161	1.368
Affected by the NCDP policy	1.370	***	0.057	1.262	1.487
Information about clinical safety and efficacy of generic drugs					
No information available	Reference				
Insufficient information	1.168	***	0.049	1.076	1.267
Sufficient information	4.197	***	0.183	3.852	4.573
Reimbursement rates of originator drugs					
80%	Reference				
50%	0.997		0.042	0.918	1.082
20%	1.367	***	0.058	1.259	1.485
Years of clinical practice					
≤10	Reference				
(10, 20]	1.536	*	0.311	1.032	2.285
>20	2.740	***	0.743	1.610	4.663
Highest education attainment of physicians					
Undergraduate and below	Reference				
Master	0.620	*	0.118	0.426	0.902
PhD	0.545	**	0.123	0.350	0.849
Gender					
Female	Reference				
Male	1.363		0.229	0.981	1.893

(Note)*P ≤ 0.05, **P ≤ 0.01, ***P ≤ 0.001, Originator choice = 0, High-quality generic choice =1

S4. Subgroup analysis of illness severity of physicians' preferences for prescribing originator and its generic drugs

Subgroup analysis C	Odds Ratio	P	Std. Err.	95% Conf.Interval	
Severity of illness					
Mild and moderate disease	Reference				
Severe disease	0.070	***	0.004	0.063	0.078
Price ratio of generics vs. originator drugs					
1:2	Reference				
3:10	1.220	***	0.070	1.090	1.364
1:10	1.177	**	0.067	1.052	1.316
Hospital cost control constraints targeting originator drugs					
Not restricted by hospital cost-controls	Reference				
Affected by hospital cost-control measures	1.150	*	0.066	1.028	1.287
Affected by the NCDP policy	1.444	***	0.083	1.291	1.616
Information about clinical safety and efficacy of generic drugs					
No information available	Reference				
Insufficient information	1.139	*	0.066	1.017	1.275
Sufficient information	4.955	***	0.300	4.400	5.579
Reimbursement rates of originator drugs					
80%	Reference				
50%	0.977		0.056	0.873	1.093
20%	1.271	***	0.073	1.136	1.422
Years of clinical practice					
≤10	Reference				
(10, 20]	1.764	*	0.465	1.052	2.956
>20	4.003	***	1.417	2.001	8.010
Highest education attainment of physicians					
Undergraduate and below	Reference				
Master	0.315	***	0.078	0.194	0.510
PhD	1.035		0.310	0.576	1.860
Gender					
Female	Reference				
Male	0.962		0.211	0.635	1.488

(Note)*P ≤ 0.05, **P ≤ 0.01, ***P ≤ 0.001, Originator choice = 0, High-quality generic choice =1

Table S5 Analysis of interact effect of physicians' preferences for prescribing originator and its generic drugs

Interact effect	Odds Ratio	P	Std. Err.	95% Conf.Interval	
Hospital cost control constraints targeting originator drugs * Price ratio of generics vs. originator drugs	1.039		0.025	0.991	1.089
Hospital cost control constraints targeting originator drugs * Reimbursement rates of originator drugs	1.064	***	0.006	1.052	1.077
Hospital cost control constraints targeting originator drugs * Years of clinical practice	1.014		0.018	0.980	1.050
Hospital cost control constraints targeting originator drugs * Highest education attainment of physicians	1.066	***	0.017	1.033	1.100
Information about clinical safety and efficacy of generic drugs * Price ratio of generics vs. originator drugs	1.283	***	0.031	1.224	1.346
Information about clinical safety and efficacy of generic drugs * Reimbursement rates of originator drugs	1.064	***	0.006	1.052	1.077
Information about clinical safety and efficacy of generic drugs * Years of clinical practice	0.889	***	0.016	0.859	0.922
Information about clinical safety and efficacy of generic drugs * Highest education attainment of physicians	1.131	***	0.019	1.094	1.168

(Note)*P ≤ 0.05, **P ≤ 0.01, ***P ≤ 0.001, Originator choice = 0, High-quality generic choice =1

S1 Primary analysis of physicians' preferences for prescribing originator and its generic drugs

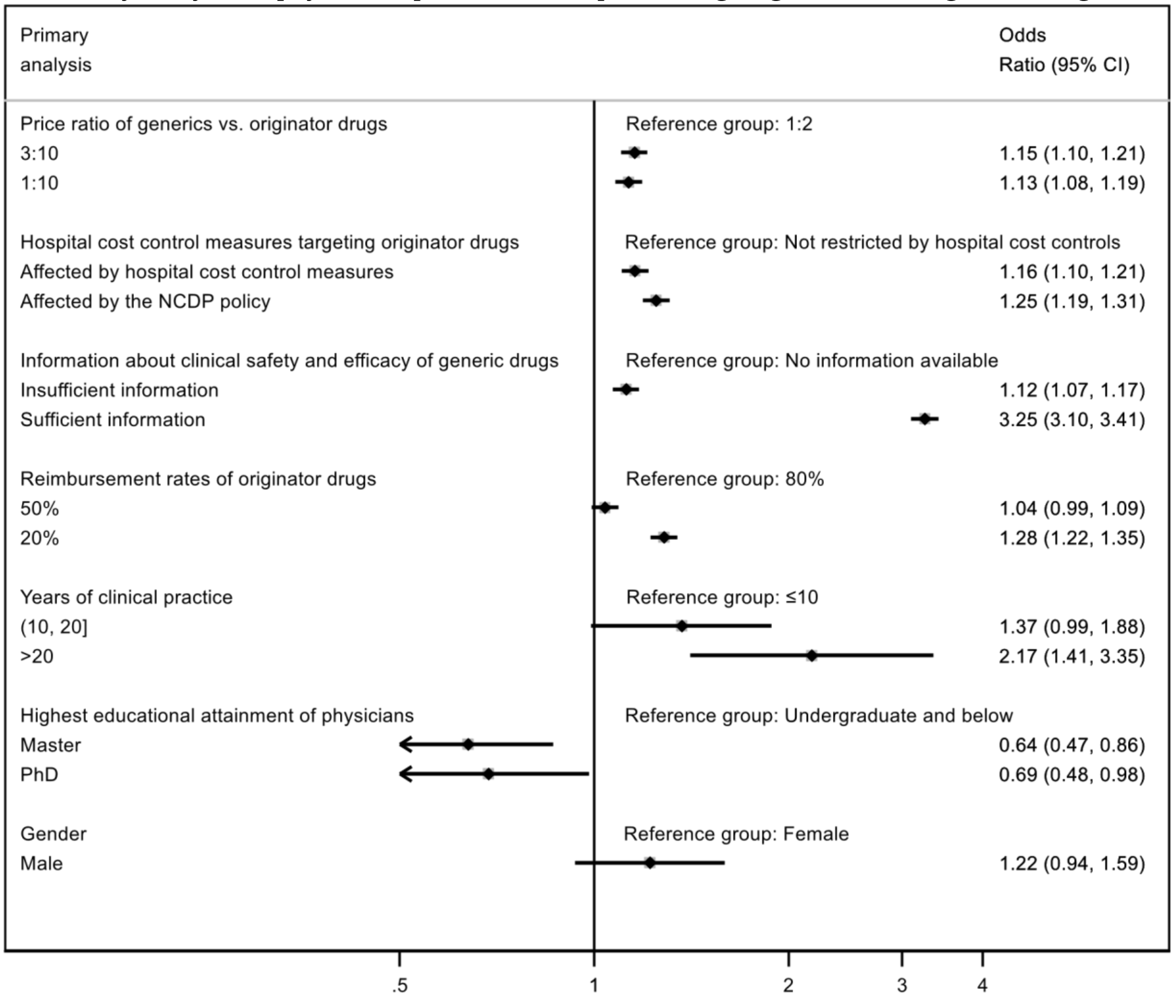

(Note) "<" represents OR < 0.5, ">" represents OR > 4

S2 Subgroup analysis of physicians' preferences for prescribing originator and its generic drugs

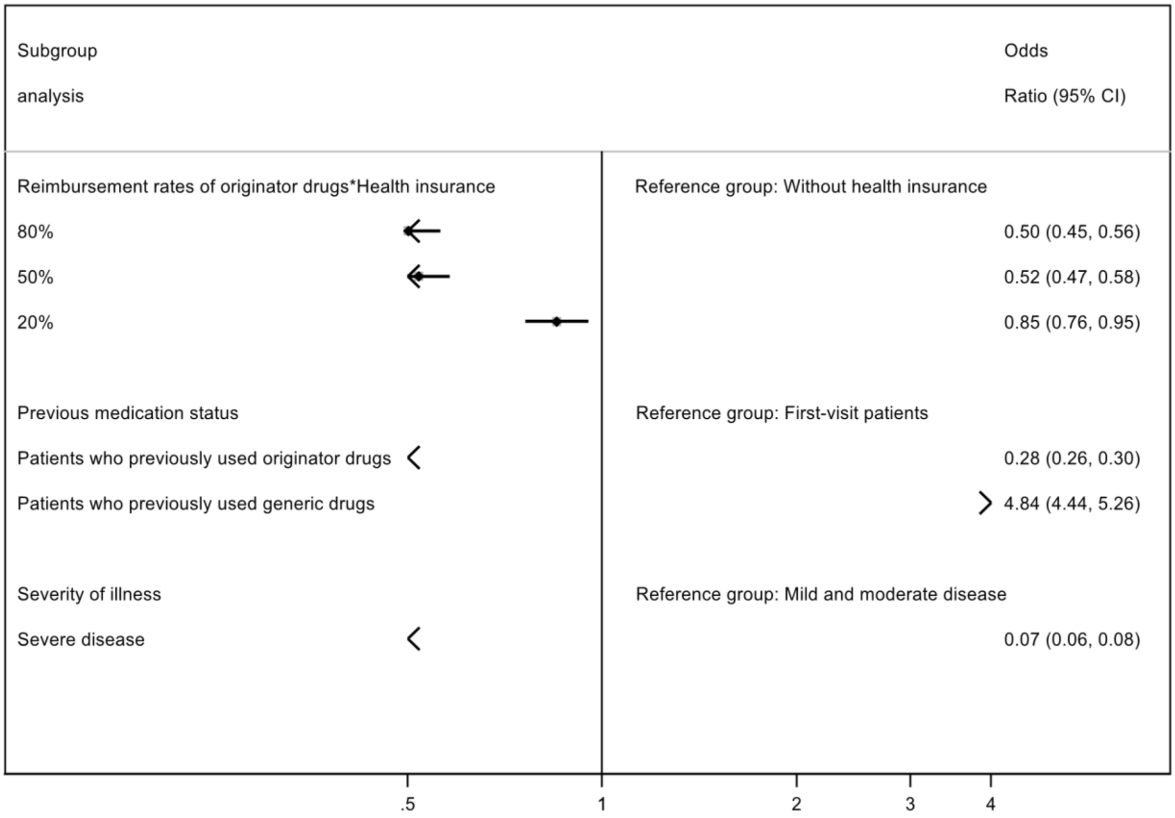

(Note) "<" represents OR < 0.5, ">" represents OR > 4

S3 Subgroup analysis of health insurance status of physicians' preferences for prescribing originator and its generic drugs

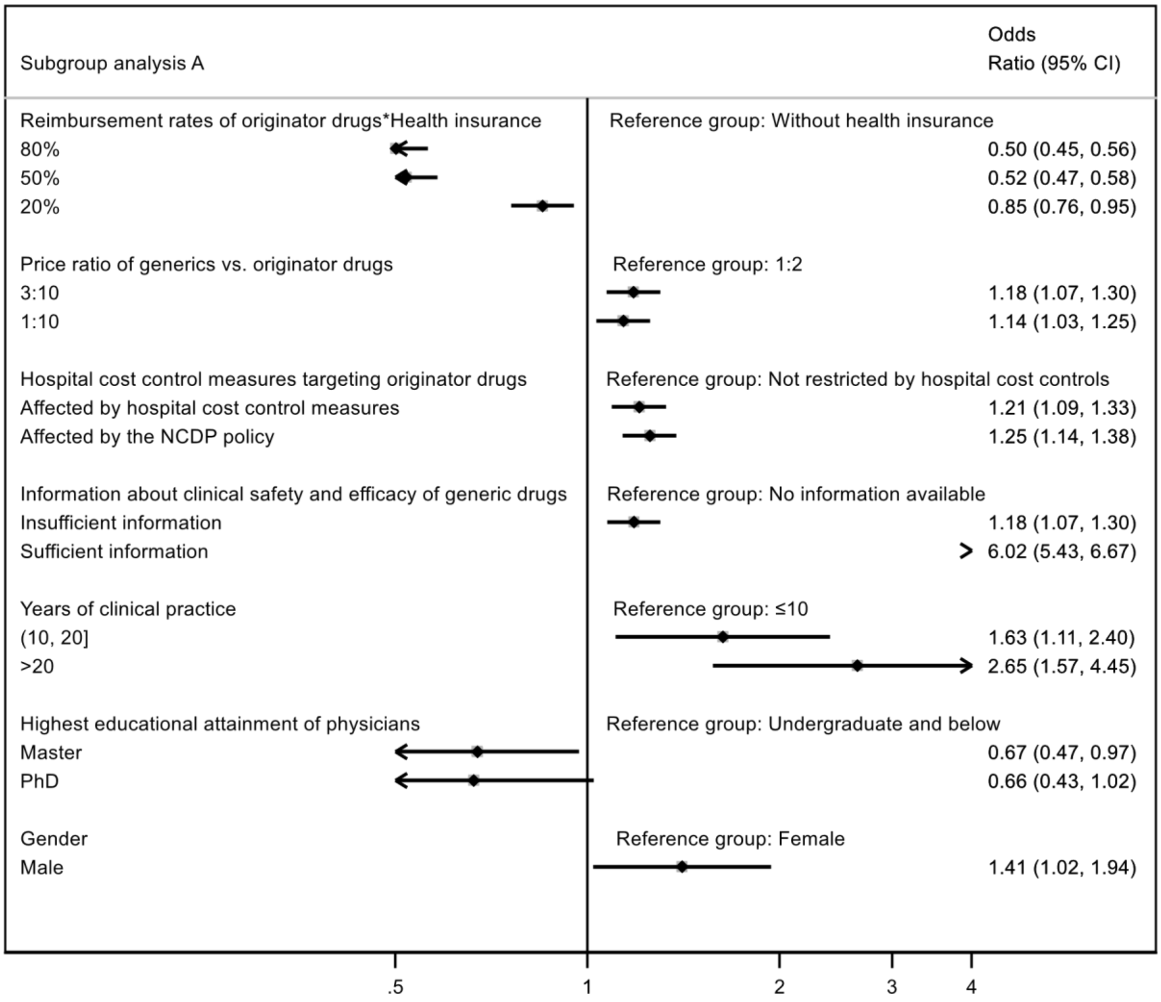

(Note) "<" represents OR < 0.5, ">" represents OR > 4

S4 Subgroup analysis of previous medication status of physicians' preferences for prescribing originator and its generic drugs

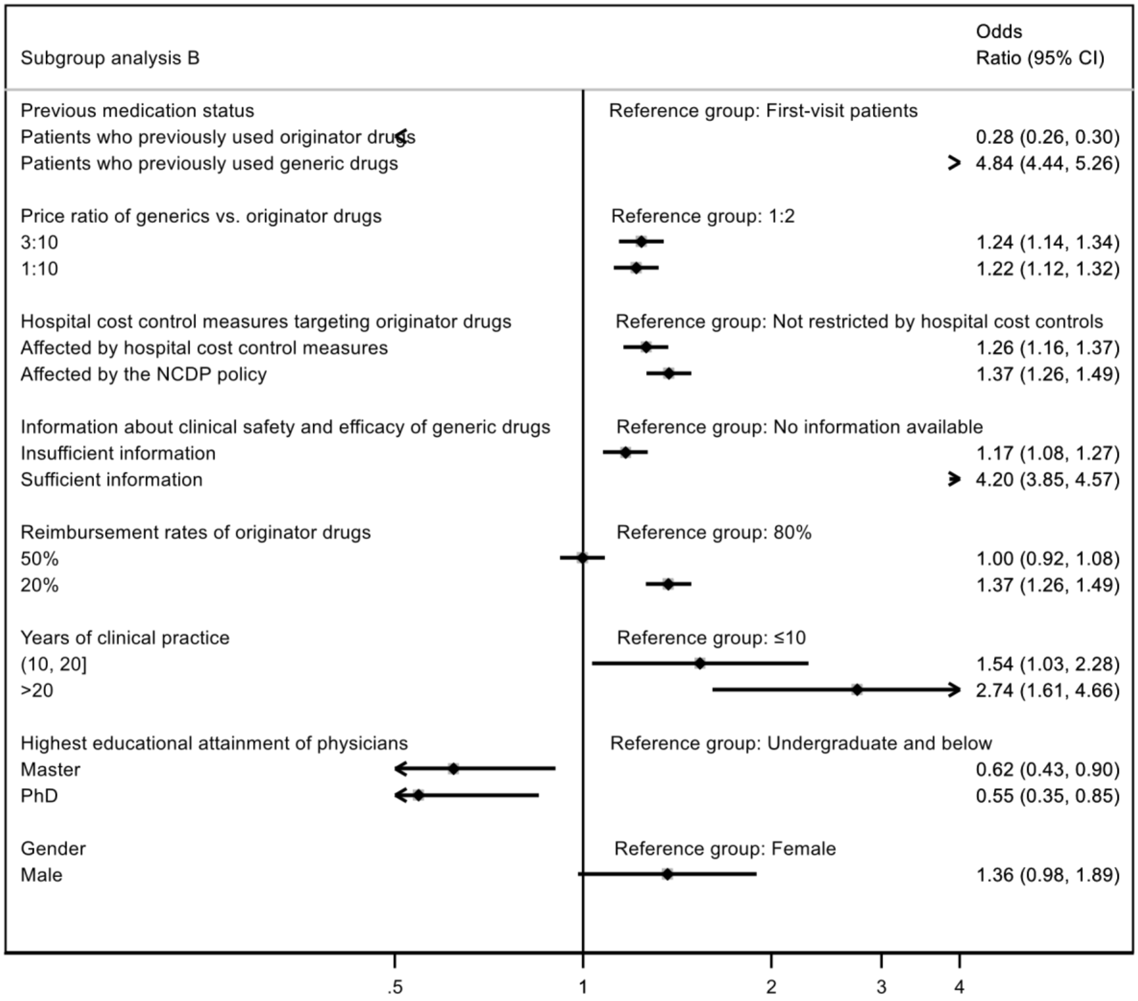

(Note) "<" represents OR < 0.5, ">" represents OR > 4

S5 Subgroup analysis of illness severity of physicians' preferences for prescribing originator and its generic drugs

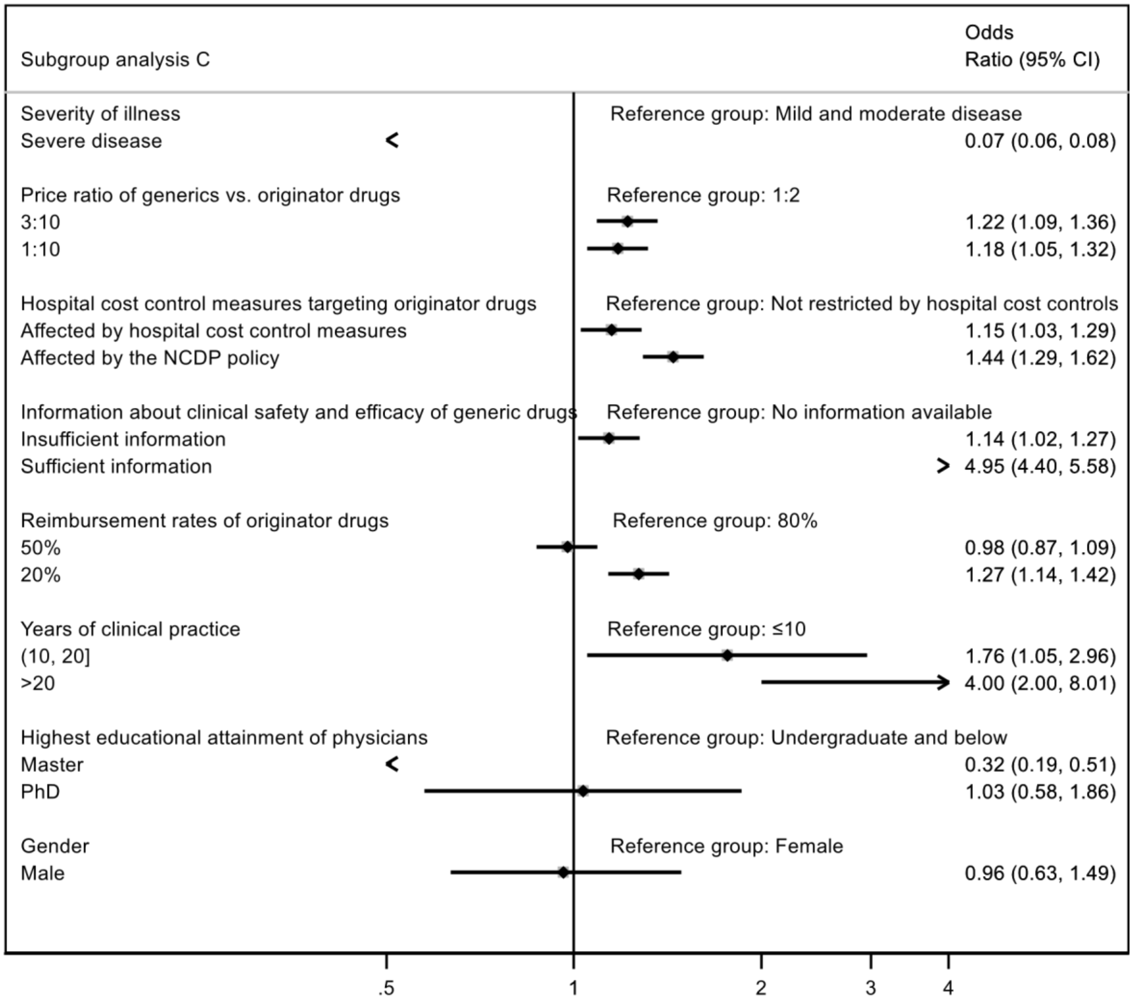

(Note) "<" represents OR < 0.5, ">" represents OR > 4

S6 Analysis of interact effect of physicians' preferences for prescribing originator and its generic drugs

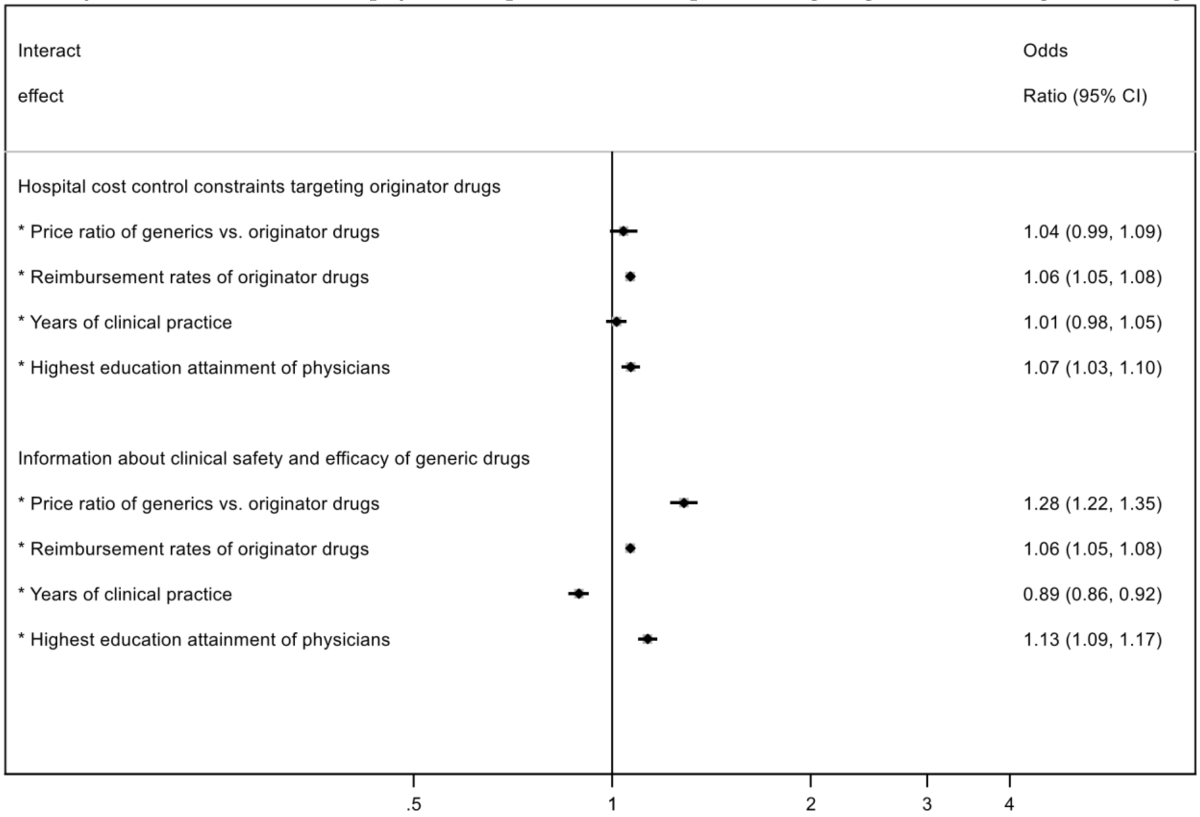

Supplement: Supplementary file 1 — contains Tables S1-S5 and Figures S1-S5. [file ijhpm-13-8392-s001.pdf]
