# Supplementary material for: Chinese Physicians’ Preference for Prescribing Brand-Name vs. Generic: A Discrete Choice Experiment
Source: Int J Health Policy Manag. 2024 Dec 29;13:8392. doi: 10.34172/ijhpm.8392 (PMC11806230; doi:10.34172/ijhpm.8392)
Supplement: Supplementary file 3 — The Questionnaire of Our Study. [file ijhpm-13-8392-s003.pdf]

Article title: Chinese Physicians' Preference for Prescribing Brand-Name vs. Generic: A Discrete Choice Experiment

Journal name: International Journal of Health Policy and Management (IJHPM)

Authors' information: Ruilin Wang[1]¶, Zhiyuan Wang[1]¶, Xiaoyu Li[1], Lin Bai[1], Pingan Fan[1], Huangqianyu Li[2], Yuanyuan Tang[3], Xin Li[4], Yangmu Huang[5], Xiaoyan Nie[1,2], Luwen Shi[1,2], Jing Chen[1,2]*

[1]Department of Pharmacy Administration and Clinical Pharmacy, School of Pharmaceutical Sciences, Peking University, Beijing, China.

[2]International Research Center for Medicinal Administration, Peking University, Beijing, China.

[3]Bidding Management Office, Suqian First Hospital, Suqian, China.

[4]Department of Clinical Pharmacy, School of Pharmacy, Nanjing Medical University, Nanjing, China.

[5]School of Public Health, Peking University, Beijing, China.

***Correspondence to:** Jing Chen; Email: jingchen@bjmu.edu.cn

¶ Both authors contributed equally to this paper.

Citation: Wang R, Wang Z, Li X, et al. Chinese physicians' preference for prescribing brand-name vs. generic: a discrete choice experiment. Int J Health Policy Manag. 2024;13:8392. doi:10.34172/ijhpm.8392

Supplementary file 3. The Questionnaire of Our Study

Dear Doctor,

We are a research team from Peking University and Nanjing Medical University, funded by the National Natural Science Foundation of China, conducting a study on "Factors influencing physicians' prescribing behavior based on the perspective of generic vs. originator drug selection". We solemnly promise that this survey is for academic research only and will not be used for other purposes. All information collected during the survey will be kept absolutely confidential and kept by the surveyor. This survey is completely voluntary, if you know and agree with the above instructions, please continue to fill out the survey. Thanks for your cooperation.

Peking University

September 2019

Signature:

Part 1: Basic characteristics

Q1. What is your gender?

□Male □Female

Q2. What is your education level?

□Undergraduate and below □Master □PhD

Q3. What is your health care facility? ___________________

Q4. What is the level of health care facility?

□Tertiary Hospital □Secondary Hospital □Primary health care facilities

Q5. How many years have you been in clinical practice?

□1-10 years □11-20 years □21-30 years □＞30 years

Part 2: 9 selection cards for different scenarios simulated according to drug characteristics and prices, please compare and choose carefully.

PS: The first item of each selection card is the type of drug: one originator drug (DRUG A), and one high-quality generic drug (DRUG B), please select one drug you intend to prescribe for the patient in the following scenarios.

Scenario 1:

Scenario characteristics	DRUG A	DRUG B
Drug	Originator Drug	High-quality Generic Drug
Drug price	50 yuan	25 yuan
Reimbursement rate	50%	90%
Hospital cost control constraints	Not restricted by hospital cost controls	Not restricted by hospital cost controls
Information on clinical safety and efficacy	Lots of information on clinical safety and efficacy	Lots of information on clinical safety and efficacy

When you meet the following patients, your choices are:

Without health insurance: □DRUG A □DRUG B

With health insurance: □DRUG A □DRUG B

When you meet the following patients, your choices are:

First-visit patients: □DRUG A □DRUG B

Patients who previously used originator drugs: □DRUG A □DRUG B

Patients who previously used generic drugs: □DRUG A □DRUG B

When you meet the patients with following condition, your choices are:

DRUG A

Drug	Originator Drug	High-quality Generic Drug
Scenario characteristics		DRUG B
Drug price	50 yuan	
Reimbursement rate		
Hospital cost control		Not restricted by hospital cost

Severe disease: □DRUG A □DRUG B

Mild and moderate disease: □DRUG A □DRUG B

Scenario 2:

15 yuan

50% 90%

Affected by hospital cost control

constraints controls

Information on clinical Lots of information on clinical No clinical safety and efficacy safety and

efficacy safety and efficacy information available

When you meet the following patients, your choices are:

Without health insurance: □DRUG A □DRUG B

With health insurance: □DRUG A □DRUG B

When you meet the following patients, your choices are:

First-visit patients: □DRUG A □DRUG B

Patients who previously used originator drugs: □DRUG A □DRUG B

Patients who previously used generic drugs: □DRUG A □DRUG B

When you meet the patients with following condition, your choices are:

Severe disease: □DRUG A □DRUG B

Mild and moderate disease: □DRUG A □DRUG B

Scenario 3:

Scenario characteristics	DRUG A	DRUG B
Drug	Originator Drug	High-quality Generic Drug
Drug price	50 yuan	5 yuan
Reimbursement rate	20%	90%

Hospital cost control constraints	Affected by hospital cost control	Not restricted by hospital cost controls
Information on clinical safety and efficacy	Lots of information on clinical safety and efficacy	Lots of information on clinical safety and efficacy

When you meet the following patients, your choices are:

Without health insurance: □DRUG A □DRUG B

With health insurance: □DRUG A □DRUG B

When you meet the following patients, your choices are:

Scenario 4:

Scenario characteristics	DRUG A	DRUG B
Drug	Originator Drug	High-quality Generic Drug
Drug price	50 yuan	15 yuan
Reimbursement rate	80%	90%
Hospital cost control constraints	Affected by NCDP policy	Not restricted by hospital cost controls
Information on clinical safety and efficacy	Lots of information on clinical safety and efficacy	Lots of information on clinical safety and efficacy

When you meet the following patients, your choices are:

Without health insurance: □DRUG A □DRUG B

With health insurance: □DRUG A □DRUG B

When you meet the following patients, your choices are:

First-visit patients: □DRUG A □DRUG B

Patients who previously used originator drugs: □DRUG A □DRUG B

Patients who previously used generic drugs: □DRUG A □DRUG B

When you meet the patients with following condition, your choices are:

Severe disease: □DRUG A □DRUG B

Mild and moderate disease: □DRUG A □DRUG B

Scenario 5:

Scenario characteristics	DRUG A	DRUG B
Drug	Originator Drug	High-quality Generic Drug
Drug price	50 yuan	15 yuan
Reimbursement rate	20%	90%
Hospital cost control constraints	Not restricted by hospital cost controls	Not restricted by hospital cost controls

Scenario characteristics	DRUG A	DRUG B
Drug	Originator Drug	High-quality Generic Drug
Drug price	50 yuan	
Reimbursement rate	80%	
Hospital cost control		Not restricted by hospital cost
Information on clinical safety and efficacy	Lots of information on clinical safety and efficacy	Less information on clinical safety and efficacy

When you meet the following patients, your choices are:

Without health insurance: □DRUG A □DRUG B

With health insurance: □DRUG A □DRUG B

When you meet the following patients, your choices are:

Scenario 6:

25 yuan

90%

Affected by hospital cost control

constraints controls

Information on clinical Lots of information on clinical Less information on clinical safety and efficacy safety and efficacy safety and efficacy

When you meet the following patients, your choices are:

Without health insurance: □DRUG A □DRUG B

With health insurance: □DRUG A □DRUG B When you meet the following patients, your choices are:

First-visit patients: □DRUG A □DRUG B

Patients who previously used originator drugs: □DRUG A □DRUG B

Patients who previously used generic drugs: □DRUG A □DRUG B

When you meet the patients with following condition, your choices are:

Severe disease: □DRUG A □DRUG B

Mild and moderate disease: □DRUG A □DRUG B

Scenario 7:

Scenario characteristics	DRUG A	DRUG B
Drug	Originator Drug	High-quality Generic Drug
Drug price	50 yuan	
Reimbursement rate	80%	
Hospital cost control		Not restricted by hospital cost

Scenario characteristics	DRUG A	DRUG B
Drug	Originator Drug	High-quality Generic Drug
Drug price	50 yuan	25 yuan
Reimbursement rate	20%	90%
Hospital cost control constraints	Affected by NCDP policy	Not restricted by hospital cost controls
Information on clinical safety and efficacy	Lots of information on clinical safety and efficacy	No clinical safety and efficacy information available

When you meet the following patients, your choices are:

Without health insurance: □DRUG A □DRUG B

With health insurance: □DRUG A □DRUG B

When you meet the following patients, your choices are:

Scenario 8:

5 yuan

90%

Not restricted by hospital cost

constraints controls controls

Information on clinical Lots of information on clinical No clinical safety and efficacy safety

and efficacy safety and efficacy information available

When you meet the following patients, your choices are:

Without health insurance: □DRUG A □DRUG B

With health insurance: □DRUG A □DRUG B When you

meet the following patients, your choices are:

First-visit patients: □DRUG A □DRUG B

Patients who previously used originator drugs: □DRUG A □DRUG B

Patients who previously used generic drugs: □DRUG A □DRUG B

When you meet the patients with following condition, your choices are:

Scenario characteristics	DRUG A	DRUG B
Drug	Originator Drug	High-quality Generic Drug
Drug price	50 yuan	
Reimbursement rate	80%	
Hospital cost control		Not restricted by hospital cost

Severe disease: □DRUG A □DRUG B

Mild and moderate disease: □DRUG A □DRUG B

Scenario 9:

Scenario characteristics	DRUG A	DRUG B
Drug	Originator Drug	High-quality Generic Drug
Drug price	50 yuan	5 yuan
Reimbursement rate	50%	90%
Hospital cost control constraints	Affected by NCDP policy	Not restricted by hospital cost controls
Information on clinical safety and efficacy	Lots of information on clinical safety and efficacy	Less information on clinical safety and efficacy

When you meet the following patients, your choices are:

Without health insurance: □DRUG A □DRUG B

With health insurance: □DRUG A □DRUG B When you

meet the following patients, your choices are:

First-visit patients: □DRUG A □DRUG B

Patients who previously used originator drugs: □DRUG A □DRUG B

Patients who previously used generic drugs: □DRUG A □DRUG B

When you meet the patients with following , your choices are:

Severe disease: □DRUG A □DRUG B

Mild and moderate disease

Thank you for filling out this survey!
